# Cancer During Pregnancy: Navigating Clinical and Research Challenges

**DOI:** 10.3390/curroncol33050296

**Published:** 2026-05-19

**Authors:** Mackenzie K. Callaway, Lizelle Comfort, Dhivyaa Anandan, Ruby Sharma, Narjust Florez, Traci R. Lyons, Doris Germain, Kathleen R. Cho, Burton L. Rochelson, Clarissa Bonanno, Kutluk Oktay, Sudarshana Roychoudhury, Eileen O’donnell, Richard Barakat, Joanne Marquardt, Diana W. Bianchi, Elyce Cardonick, Larry Norton, Ann H. Partridge, Susan M. Domchek, Virginia F. Borges, Frédéric Amant, Camila O. Dos Santos

**Affiliations:** 1 Cancer Center, Cold Spring Harbor Laboratory, Cold Spring Harbor, NY 11724, USA; callaway@cshl.edu (M.K.C.); anandan@cshl.edu (D.A.); 2 Department of Obstetrics and Gynecology, Division of Maternal Fetal Medicine, Northwell Health and Donald and Barbara Zucker School of Medicine at Hofstra/Northwell, Northwell Health, New Hyde Park, NY 11042, USA; lcomfort@northwell.edu (L.C.); brochels@northwell.edu (B.L.R.); cbonanno@northwell.edu (C.B.); 3 Stony Brook Medical Scientist Training Program, Renaissance School of Medicine at Stony Brook University, Stony Brook, NY 11790, USA; 4 Northwell Health Cancer Institute, 1111 Marcus Avenue, New Hyde Park, NY 11042, USA; rsharma5@northwell.edu (R.S.); eodonnell1@northwell.edu (E.O.); rbarakat@northwell.edu (R.B.); 5 Dana-Farber Cancer Institute, Harvard Medical School, Boston, MA 02215, USA; narjust_florez@dfci.harvard.edu (N.F.); ann_partridge@dfci.harvard.edu (A.H.P.); 6 Department of Medicine, Division of Medical Oncology, University of Colorado Anschutz Medical Campus, Aurora, CO 80045, USA; traci.lyons@cuanschutz.edu (T.R.L.); virginia.borges@cuanschutz.edu (V.F.B.); 7 University of Colorado Cancer Center Young Women’s Breast Cancer Translational Program, Aurora, CO 80045, USA; 8 Tisch Cancer Institute, Department of Medicine, Icahn School of Medicine at Mount Sinai, New York, NY 10029, USA; doris.germain@mssm.edu; 9 Department of Pathology, The University of Michigan Medical School, Ann Arbor, MI 48109, USA; kathcho@med.umich.edu; 10 Department of Obstetrics, Gynecology and Reproductive Sciences, Yale School of Medicine, New Haven, CT 06510, USA; kutluk.oktay@yale.edu; 11 Innovation Institute for Fertility Preservation, New York, NY 10016, USA; 12 Department of Pathology and Laboratory Medicine, Donald and Barbara Zucker School of Medicine at Hofstra/Northwell Health, Greenvale, NY 11549, USA; sroychoudh@northwell.edu; 13 Babylon Breast Cancer Coalition, 218N Wellwood Ave Suite #2, Lindenhurst, NY 11757, USA; jtmarquardt@aol.com; 14 Prenatal Genomics and Therapy Section, Center for Precision Health Research, National Human Genome Research Institute, National Institutes of Health, 35A Convent Drive, Bethesda, MD 20892, USA; diana.bianchi@nih.gov; 15 Eunice Kennedy Shriver National Institute of Child Health and Human Development, National Institutes of Health, 31 Center Drive, Bethesda, MD 20892, USA; 16 Department of Obstetrics and Gynecology, Cooper University Health Care, Camden, NJ 08103, USA; cardonick-elyce@cooperhealth.edu; 17 Department of Medicine, Memorial Sloan Kettering Cancer Center, New York, NY 10065, USA; nortonl@mskcc.org; 18 Department of Medicine, University of Pennsylvania Health System, Philadelphia, PA 19104, USA; susan.domchek@pennmedicine.upenn.edu; 19 Baser Center for BRCA, Abramson Cancer Center, University of Pennsylvania, Philadelphia, PA 19104, USA; 20 Department of Oncology, KU Leuven, 3001 Leuven, Belgium; frederic.amant@uzleuven.be; 21 Department of Obstetrics and Gynecology, UZ Leuven, 3000 Leuven, Belgium; 22 Gynecologic Oncology, Antoni van Leeuwenhoek, 1066 Amsterdam, The Netherlands

**Keywords:** cancer during pregnancy, maternal–fetal care, cancer, health disparity

## Abstract

The incidence of cancer during pregnancy is rising, yet scientific understanding and clinical management remain underdeveloped. Delayed diagnoses, limited therapeutic options, and lack of safety data exacerbate the clinical challenges of treating cancer during pregnancy. Further, the biology of gestational cancers is poorly understood due to the scarcity of model systems and mechanistic studies. This manuscript presents a multidisciplinary perspective from a group of researchers and clinicians to evaluate the current state of pregnancy-associated cancers, identify unmet clinical and biological questions, and propose strategies to improve diagnosis, treatment, and maternal–fetal outcomes.

## Simple Summary

Cancer during pregnancy is becoming increasingly common, yet it remains one of the most underexplored areas in oncology, creating major challenges for timely diagnosis, safe treatment, and informed clinical decision-making. This manuscript brings together multidisciplinary experts alongside patient perspectives to examine the unique biological, clinical, and systemic barriers that have limited progress in understanding and managing cancer during pregnancy. It highlights critical gaps driven by delayed detection, overlapping pregnancy-related symptoms, limited access to evidence-based guidance, and the historical exclusion of pregnant patients from clinical trials and research studies. By defining the most urgent unanswered questions, this work aims to guide future efforts to improve early detection, optimize treatment strategies that protect both parent and fetus, and expand the development of more accurate preclinical models, registries, and patient-centered data resources. Ultimately, this perspective seeks to accelerate research, reduce uncertainty in care, and improve outcomes for pregnant patients facing cancer.

## 1. Introduction

Over recent decades, the incidence of cancer diagnosed during pregnancy has steadily increased [1]. While the basis for this rise remains incompletely understood, rates have grown from approximately 75 cases per 100,000 pregnancies in 2002 to nearly 100 per 100,000 by 2012, where they have since plateaued [2,3,4]. This trend is expected to continue, driven by increasing maternal age and the rising incidence of early-onset cancers in the general population [5]. Despite this growing prevalence, cancer diagnosis during pregnancy is frequently delayed, largely due to perceived limitations in screening strategies and the misattribution of cancer-related symptoms to normal physiological changes in pregnancy [6,7,8].

The scarcity of clinical and scientific data on cancer during pregnancy extends beyond its relatively low incidence and reflects a long-standing exclusion of women, particularly pregnant individuals, from clinical research [9,10]. Although the inclusion of women in NIH-sponsored trials was prioritized in the early 1990s, significant disparities persist [11]. Notably, only ~1% of clinical trials explicitly include pregnancy. While initiatives such as the task force on research specific to Pregnant Women and Lactating Women (PRGLAC) and the Coalition to Advance Maternal Therapeutics have begun to address this gap, pregnant and lactating individuals remain underrepresented, limiting evidence-based clinical guidance [12,13]. As a result, the management of cancer during pregnancy presents a unique clinical challenge. While several anti-cancer therapies are considered safe for use during pregnancy, decisions must balance maternal benefit with potential risks to fetal development and lactation. These complexities are further compounded by a limited understanding of the underlying biology of pregnancy-associated cancers. It remains unclear whether tumors arising during pregnancy are biologically distinct from those in non-pregnant individuals, highlighting a critical gap in knowledge [14].

These gaps in the clinical care of pregnant patients with cancer rival those of our understanding of the disease itself. The biology of cancer during pregnancy is vastly unexplored, which for years has raised questions regarding whether malignant tumors are the same as those found in non-pregnant patients [15]. Further, the lack of model systems and research focus to study cancer during pregnancy has limited the pre-clinical testing of therapeutic strategies and the balance of their efficacy in targeting cancer progression with consequences to maternal–fetal health. Additionally, the absence of pre-clinical research has stunted the development of diagnostic tools for early detection of cancers during pregnancy. Of the current literature that exists, there is a predominant focus on breast cancer during pregnancy, with a wide variety of additional, rarer malignancies being even less well-understood.

Compelled by these challenges, this manuscript integrates perspectives from an interdisciplinary group of scientists and clinicians to redefine cancer during pregnancy as both a clinical and biological frontier. We aim to synthesize current knowledge, identify key gaps, and propose strategies to improve diagnosis, management, and research prioritization. Ultimately, advancing this field will require coordinated efforts to integrate clinical data, experimental models, and translational insights to improve outcomes for both mother and child.

## 2. The Incidence and the Risk of Cancer During Pregnancy

Pregnancy induces widespread systemic changes across multiple organs, including the brain, liver, gut, heart, breast, thyroid, and kidney, and modulates immune function to support maternal–fetal tolerance [16,17,18,19]. Within this dynamic physiological context, the most commonly diagnosed cancers during pregnancy include breast, cervical, and thyroid carcinomas; melanoma; and hematological malignancies [1,20,21]. Emerging data further suggest an increasing incidence of colorectal cancer, certain lung cancer subtypes, and choriocarcinoma in pregnant populations [22,23].

Importantly, cancer diagnoses during pregnancy are often made weeks to months after tumor initiation, complicating efforts to disentangle pregnancy-driven biological effects from pre-existing disease processes. Among known risk factors, advanced maternal age remains the most significant contributor across cancer types [1,24]. Importantly, while pregnancy and cancer may temporally coincide, critical unanswered questions remain regarding whether pregnancy-associated physiological remodeling and/or underlying genomic predispositions function as independent, convergent, or synergistic drivers of tumor initiation, acceleration, progression, or metastatic potential, underscoring the urgent need to define the molecular mechanisms through which reproductive physiology may alter cancer emergence, exacerbate disease course, and ultimately complicate translational management and therapeutic decision-making.

Race and ethnicity represent additional, yet underexplored, dimensions of risk [20,25]. While their roles in cancer during pregnancy are not well characterized, disparities in cancer outcomes and maternal mortality in the general population suggest important intersections with social determinants of health [26,27,28,29]. The underrepresentation of diverse populations in clinical research further underscores the need for globally coordinated registries and studies addressing these disparities [30]. Though the lack of genomic studies of tumors from pregnant patients raises uncertainty about the specific association of genomic alterations with cancer onset during pregnancy, large clinical studies have shed light on the expression of genes that define breast cancer associated with pregnancy (gestational and postpartum), thus supporting the notion that cancers during pregnancy may exhibit distinct molecular programs [31].

It also remains unclear whether genetic predisposition confers additional, pregnancy-specific cancer risk, as this relationship is likely confounded by the temporal overlap between reproductive years and the peak incidence of early-onset cancers [32,33]. Specific to breast cancer patients, however, a previous diagnosis of cancer does not confer additional risk of new cancers or recurrences developing during or after pregnancy, at least in the short term, suggesting that pregnancy after a cancer diagnosis is relatively safe but must be examined on a case-by-case basis [34,35].

***Recommendation corner:*** There is urgent consensus on the need for large-scale, multi-center, and globally coordinated studies to establish comprehensive datasets of pregnant patients with cancer. These efforts should integrate clinical, demographic, and molecular data, including tumor subtype, stage, pregnancy characteristics, and long-term maternal and offspring outcomes, to improve risk stratification and early detection. From a research perspective, defining the molecular and cellular mechanisms underpinning pregnancy-associated cancers is essential. Understanding how pregnancy-induced physiological changes reshape tissue homeostasis and tumor biology will be critical to determining whether these cancers represent a distinct disease entity and to guiding the development of targeted diagnostic and therapeutic strategies.

## 3. Cancer Screening and Diagnosis During Pregnancy

In non-pregnant individuals, symptoms suggestive of cancer development typically prompt timely diagnostic evaluation. In contrast, similar symptoms arising during pregnancy are often attributed to normal physiological changes associated with gestation, which can delay clinical suspicion and workup. As a result, pregnant patients presenting with cancer-related symptoms may experience significant delays in diagnosis, as common warning signs, such as pain perception, respiratory function, and gastrointestinal activity, overlap with expected features of pregnancy. This diagnostic ambiguity underscores the need for heightened clinical vigilance and refined guidelines to distinguish benign pregnancy-related symptoms from early manifestations of malignancy [36] (Table 1). Indeed, studies report significantly higher rates of delayed breast cancer diagnosis in pregnant and postpartum women compared to non-pregnant individuals. These delays are often reinforced by both clinical and patient-level misinterpretation of symptoms.

For individuals at elevated risk, such as those with a strong family history or known genetic predisposition, enhanced surveillance strategies, including genomic testing and targeted imaging, may be warranted prior to symptom onset. For example, carriers of pathogenic BRCA1/2 variants may undergo regular mammographic screening to enable early detection, followed by additional imaging or biopsy when clinically indicated. In such high-risk settings, genetic counseling and consideration of cascade screening for biological relatives may further support risk assessment and early intervention.

One retrospective study found that breast cancer diagnosis was disproportionately delayed in pregnant and postpartum women (60%) compared to a nonpregnant cohort (0%) [55]. Anecdotal commentary by survivors of cancer during pregnancy underscored similar experiences in which patients exhibited persistent and severe symptoms that were initially attributed to pregnancy but were ultimately signs of cancer (see the ***Cancer Advocate Perspective***). Therefore, future updates in the diagnosis of cancer during pregnancy should address the need to refine detection strategies, address limitations of conventional tools, and ensure the safe application of standard diagnostic platforms in pregnant patients. Improved clinical guidance and broader awareness of how to effectively and safely implement imaging and biomarker-based assessments during pregnancy are essential to reduce diagnostic delays and improve outcomes for both mother and fetus.

Despite these challenges, a range of diagnostic modalities can be safely and effectively employed during pregnancy when appropriately selected:

**Breast Imaging:** Contrary to common belief, mammography is not contraindicated during pregnancy [56]. However, milk production can obscure tumor masses, limiting its sensitivity [56,57]. In these cases, ultrasound, already routinely used during pregnancy to monitor fetal development, serves as a valuable adjunct approach for breast cancer detection, aiding in identifying malignancies in several soft tissues and abdominal organs [58].

**Whole-body MRI:** Similarly, during pregnancy, imaging can be safely performed using whole-body MRI, a non-ionizing modality that minimizes fetal risk and does not require gadolinium contrast [59]. Interestingly, manganese-rich fruit juices, such as pineapple juice, have been used as safe alternative contrast agents in patients with suspected gastrointestinal and reproductive malignancies who cannot receive gadolinium [60]. Incorporating similar safe and reliable alternatives may be critical in improving cancer detection during pregnancy.

**Whole-body PET/CT:** For some malignancies, PET/CT may be the preferred diagnostic modality, such as for suspected thoracic malignancy or in the case of metastatic disease not otherwise detected on MRI [61]. Because these techniques involve ionizing radiation, which may pose fetal risk at doses exceeding recommended thresholds, their use should be restricted to diagnostic procedures that inform clinical management, ensuring cumulative fetal exposure remains below 100 mGy [62]. Although abdominal shielding has traditionally been used during ionizing radiation procedures in pregnant patients (such as CT or X-ray imaging), it offers no proven benefit in protecting the fetus and may inadvertently increase fetal radiation exposure [63].

**Biomarkers:** Serum and blood-based biomarkers are potential, non-invasive tools for cancer detection and monitoring. Yet, many commonly used solid tumor markers, such as Cancer Antigen 15-3 (CA 15-3, breast cancer), Squamous Cell Carcinoma Antigen (SCCA, squamous cell carcinomas), Cancer Antigen 125 (CA-125, ovarian cancer), Human Chorionic Gonadotropin (hCG, choriocarcinoma), and Alpha-fetoprotein (AFP, liver and ovarian cancers), are physiologically elevated during pregnancy and therefore have limited utility for cancer detection in this context [64,65,66]. An emerging area of research is the incidental detection of cancer in pregnant women via plasma cell-free DNA (cfDNA) sequencing, a test commonly used to screen for fetal chromosomal abnormalities [67]. In fact, in a recent prospective study, 48% of healthy pregnant women with inconclusive or non-reportable cfDNA results went on to receive a cancer diagnosis (most commonly lymphoma, colorectal, and breast malignancies) with a mean gestational age of 22 weeks at diagnosis [68]. These findings demonstrate that cancer may be more prevalent amongst asymptomatic pregnant individuals than previously recognized, highlighting a potential window for early detection.

Notably, many cancers diagnosed during pregnancy occur in otherwise asymptomatic patients, with over half of genitourinary malignancies presenting without symptoms [69]. This highlights a critical opportunity to recognize warning signs commonly misattributed to pregnancy, identify additional screening indicators, and develop alternative detection strategies.

***Recommendation corner:*** Despite ongoing advances in cancer detection, concerns about the safety of diagnostic modalities during pregnancy persist, particularly regarding potential effects on fetal development. While alternative MRI contrast approaches, such as manganese-rich oral agents, offer promising low-risk options, their broader clinical adoption will require increased provider awareness and standardized implementation. Although iodinated and gadolinium-based contrast agents are not teratogenic in animal models [70], their placental transfer warrants careful consideration before use in pregnant patients. Similarly, radioactive tracers used for procedures such as sentinel lymph node biopsy are associated with minimal fetal exposure and are generally considered safe when clinically indicated [71].

From a research perspective, integrating placental assessments into pregnancy-specific cancer models will be critical to determine the extent of fetal exposure to diagnostic interventions and to define potential downstream effects on fetal development. In parallel, the development of robust model systems and comprehensive genomic datasets will provide a foundation for identifying biomarkers suitable for non-invasive detection strategies, including liquid biopsy approaches. Equally important is the need to empower patients, advocates, and healthcare providers with clear, evidence-based information to support informed decision-making, particularly in interpreting cancer-related symptoms during pregnancy and navigating inconclusive or abnormal findings from prenatal screening tools, such as cell-free DNA assays.

## 4. Cancer Management During Gestation and the Postpartum Period

Treating cancer during pregnancy presents complex and highly nuanced clinical challenges. Historically, management strategies often favored pregnancy termination, delay of maternal therapy, or iatrogenic preterm delivery, approaches that are no longer supported by current evidence-based guidelines [72]. The adoption of more standardized, evidence-driven care has been facilitated by increased access to international advisory networks, centralized multidisciplinary care models, and sustained efforts to translate emerging scientific insights into clinical practice [73].

Despite these advances, the rarity of cancer during pregnancy continues to limit provider experience and confidence, contributing to hesitancy in the use of standard anti-cancer therapies. This uncertainty is often driven by concerns regarding treatment-related toxicities, pregnancy-specific physiological considerations, and the complexities of balancing maternal and fetal health [74]. Patients, likewise, may be reluctant to initiate therapy, even in the context of growing evidence supporting the safety and efficacy of many treatments during pregnancy [75]. Such hesitancy is frequently rooted in fears of fetal harm and persistent misconceptions that pregnancy termination or early delivery improves maternal outcomes, assumptions not supported by current clinical data.

Addressing these barriers will require not only ensuring access to expert consultation and multidisciplinary care but also improving the dissemination of clear, evidence-based information to patients and providers alike. As presented in Table 2 [76], the importance of delivering medically integrated care through multidisciplinary collaboration amongst oncologists, obstetricians, maternal–fetal medicine specialists, psychologists, lactation consultants, reproductive endocrinologists, and neonatal professionals should be in place to support medical providers and patient care teams in navigating cancer treatment during pregnancy.

Beyond delivery, however, significant gaps remain in our understanding of postpartum cancer management and long-term follow-up. There is a clear need to implement structured strategies for data collection, lactation guidance, and coordinated post-treatment care. In general, postpartum management should align with standard oncologic practices, with appropriate adjustments to account for lactation and recovery (Table 2). Importantly, postpartum breast cancers, defined as those diagnosed within ten years of childbirth, are associated with more aggressive disease and poorer outcomes [103], underscoring the urgency for focused investigation in this window of risk. Addressing these challenges will require sustained efforts to develop model systems that faithfully recapitulate cancer initiation and progression during and after pregnancy, alongside clinical studies aimed at identifying effective intervention strategies. Such approaches will be critical to advancing our understanding of how pregnancy and the postpartum state shape tumor biology and influence maternal–fetal health outcomes.

Equally important is the continued dissemination of evidence-based guidelines on treatment, delivery planning, and patient management to both clinicians and patients in order to reduce misinformation and improve quality of care. Clinical centers should also prioritize the establishment of standardized biobanking protocols, including collection of placenta, umbilical cord, maternal plasma, and amniotic fluid, in cases of suspected or confirmed cancer during pregnancy. Coupled with longitudinal follow-up of both mother and child, these efforts will provide a foundational resource to define disease etiology, assess treatment-related effects, and ultimately inform safer and more effective care strategies.

***Recommendation corner:*** A key priority is to strengthen connectivity between clinical centers caring for pregnant patients with cancer and established international networks such as the Advisory Board on Cancer, Infertility and Pregnancy (ABCIP) and the International Network on Cancer, Infertility and Pregnancy (INCIP). Engagement with these consortia enables access to up-to-date best practices, facilitates participation in expert forums, and helps overcome the inherent challenges of studying rare conditions through global data sharing and coordinated care strategies, ultimately improving both maternal and fetal outcomes. Additional efforts should include the development of dedicated support systems, such as patient-focused hotlines, and the routine use of expert consultation when clinical uncertainty arises.

Equally critical is the integration of comprehensive fertility counseling into clinical care. Discussions should address the potential impact of chemotherapy and other treatments on reproductive capacity, as well as risks associated with future pregnancies, topics that remain underrepresented in current care paradigms. This gap reflects a broader lack of understanding of how cancer therapies influence both short- and long-term gonadal function, underscoring the need for continued research and clinical attention.

When feasible, fertility preservation strategies should be proactively incorporated into treatment planning. For example, ovarian tissue cryopreservation may be considered at the time of surgical intervention, minimizing additional procedures. For patients who will continue therapy postpartum, established options, including oocyte, embryo, and ovarian tissue cryopreservation, should be discussed prior to the initiation of chemotherapy, consistent with standard oncologic practice. Integrating these considerations into shared decision-making is essential to ensure that patients are fully informed and supported in preserving future reproductive potential.

## 5. Experiencing Cancer During Pregnancy—The Cancer Advocate Perspective

**Joanne Marquardt—Breast cancer previvor and survivor.** “I am a 20-year survivor of breast cancer, 13-year survivor of colon cancer, and have lost my maternal grandmother, mother, maternal aunt, and daughter to breast cancer. Delay in diagnosis, limited treated options and minimal attention to the collateral damage in the family and children have shaped much of my life experiences with cancer. My brother and I have lived with the long-term effects of losing our mother to pregnancy-associated breast cancer at age 5 and 8, respectively. Too often, the stresses involved lead to isolation, animosities, and dysfunction, resulting in negative effects on the children. Today, thankfully there are more treatment options and support services. As someone that has lived through many losses and devoted my life to providing support to other cancer survivors, I know that support to cancer patients and their family, either through group meetings, virtual calls, or phone chats, do make a difference. Such approaches are of special importance to those pregnant patients with cancer in rural areas where multidisciplinary teams may not be available. Of specific uniqueness to pregnant patients with cancer, the involvement of survivors and volunteer groups as part of multidisciplinary care provides an opportunity to demystify social taboos regarding treating cancer during pregnancy, while providing for a source of, ‘I understand, I’ve been through it,’ support. This should include encouragement to inform and engage with children’s teachers, guidance counselors and coaches to guide the children through their challenges.”

**Eileen O’Donnell—Non-Hodgkin’s lymphoma during pregnancy survivor.** “A cancer diagnosis during pregnancy is a life-altering event, merging the emotional turmoil of cancer with the complexities of impending motherhood. The resulting uncertainty and fear can be overwhelming. The joy and anticipation of parenthood is suddenly overshadowed by anxieties about the health of both mother and child. Physicians sometimes hesitate to share information fully, fearing they may overwhelm the patient or due to uncertainty about outcomes. This information should be presented with sensitivity, hope, and empathy, emphasizing potential treatment paths and acknowledging the individual nature of each case. Further, acknowledging the emotional and physical toll of managing both pregnancy and cancer concurrently is crucial. A primary concern for expectant mothers is the impact of treatment on their unborn child. Addressing the possibility of developmental impacts, such as cognitive delays or physical challenges, allows families to access early interventions that can significantly improve outcomes. Further, an understanding of what to expect during treatment is essential for both the patient and their family. Information about available support services, including counseling and support groups, should be readily shared, as these resources offer invaluable emotional support. Understanding the implications of a cancer diagnosis during pregnancy on future fertility is also vital. Patients need information on how treatment might affect their ability to conceive again in the future. Open dialogue about diagnosis, treatment options, risks, and available resources enables patients to make informed choices that prioritize both their health and the well-being of their child.”

## 6. Closing Remarks

This perspective integrates key insights and recommendations from an international consortium of clinicians, researchers, patient advocates, and survivors committed to improving outcomes for individuals diagnosed with cancer during or shortly after pregnancy. The goal of this forum was to define clinical and research priorities that will reshape the evolving understanding and management of Cancer During Pregnancy, as summarized in Table S1. Central to this effort is the need for sustained, longitudinal follow-up of both patients and their children, an area in which registries such as INCIP play a critical role in capturing outcomes and informing future care. Additional resources for clinicians, researchers, and advocates are provided in Table S2. Looking ahead, continued dialogue and collaboration will be essential to harmonize care, expand data sharing, and accelerate both basic and translational research aimed at improving maternal and fetal outcomes.

Recent consensus statements from the American Society of Clinical Oncology (ASCO) and the European Society of Gynaecological Oncology (ESGO) represent important advances in the clinical management of cancer during pregnancy. These guidelines emphasize standardized care pathways, multidisciplinary coordination, the relative safety of imaging and systemic therapies during gestation, and the importance of international registries to improve outcomes. Notably, they reinforce that pregnancy termination is not routinely required, that many chemotherapeutic agents can be administered safely after the first trimester, and that care coordinated through expert networks such as INCIP and ABCIP leads to improved maternal–fetal outcomes. Collectively, these efforts have been instrumental in reducing therapeutic hesitation and aligning clinical practice with current evidence.

While these society-led statements provide a critical foundation for clinical care, they are largely focused on treatment delivery, safety, and outcome standardization. In contrast, the present manuscript advances a complementary, research-driven framework by addressing the fundamental biological unknowns of pregnancy-associated cancers. Specifically, we: (i) conceptualize pregnancy and the postpartum period as dynamic states of systemic and tissue remodeling that may influence tumor initiation, immune contexture, and metastatic progression; (ii) highlight the absence of mechanistic model systems that faithfully recapitulate cancer development in this context; (iii) call for integration of reproductive biology with tumor genomics, placental biology, and longitudinal maternal–offspring studies; (iv) propose standardized biobanking of placenta, maternal plasma, and fetal-derived tissues to enable molecular interrogation of disease; and (v) underscore the importance of equity, including racial disparities and the persistent underrepresentation of pregnant individuals in clinical research.

Together, these perspectives extend beyond existing clinical frameworks to define a forward-looking agenda centered on biological discovery and translational innovation. Improving outcomes for pregnant patients with cancer will require not only harmonized clinical guidelines but also a coordinated research infrastructure capable of elucidating how pregnancy reshapes tumor biology at molecular resolution. By bridging clinical practice, patient advocacy, and mechanistic science, this work aims to catalyze the next phase of the field, shifting from a focus on treatment safety to one of precision understanding and intervention.

## Figures and Tables

**Table 1:** Overlap of pregnancy- and cancer-related symptoms across organ systems. All entries represent an original synthesis by the authors based on multiple cited studies (refs. [37,38,39,40,41,42,43,44,45,46,47,48,49,50,51,52,53]) and do not reproduce or adapt previously published tables or figures.

Organ	Pregnancy-Related Symptoms	Cancer-Related Symptoms
Skin [37,38,39]	Alterations in the size, coloration, and appearance of skin nevi; hyperpigmentation	Alterations in the size, coloration, and appearance of skin nevi; hyperpigmentation
Breast [40,41]	Increases in size, alterations in shape, color and overlying skin; increased tenderness, nodularity Accessory breast tissue can enlarge during pregnancy and should be assessed during prenatal breast exams	Increases in size, alterations in shape, color and overlying skin; increased tenderness, nodularity
Thyroid [42,43]	Becomes enlarged, increased presence and size of thyroid nodules	Becomes enlarged, increased presence and size of thyroid nodules
Colon [44,45,46]	Abdominal pain and distension, nausea and vomiting, anemia, constipation, and changes in stool composition	Abdominal pain and distension, nausea and vomiting, anemia, constipation, and changes in stool composition
Lung [47,54]	Cough, chest pain, fatigue, shortness of breath	Cough, chest pain, fatigue, shortness of breath
Cervix [48,49,50]	Vaginal discharge or bleeding, fatigue, changes in urination and bowel movements	Vaginal discharge or bleeding, fatigue, changes in urination and bowel movements Diagnosis of cervical cancer may be limited by cervical distention during pregnancy and the inability to sample the endocervix
Blood [51,52,53]	Fatigue, weakness, sweating, shortness of breath, abdominal/back pain	Fatigue, weakness, sweating, shortness of breath, abdominal/back pain

**Table 2:** The utility of and risks associated with cancer intervention strategies during pregnancy and post-partum.

Intervention	Is It Utilized During Pregnancy?	Risk to Patient	Risk to Fetus/Neonate	Clarification of Risks	Considerations and Recommendations
Surgical resection	Yes	Slight increase in risk of miscarriage under general anesthesia [77,78] Comparable outcomes to non-obstetric patients [77]	No increased risk of birth defect or teratogenicity [79] Risk of low birth weight and/or premature delivery [80]	Low birth weight and premature delivery may be due to underlying medical condition warranting surgery, rather than risk of surgery itself	Surgery can be carried out at any moment during pregnancy and should not be delayed Neonatologists should be notified if the fetus is of viable gestational age in the event of preterm delivery
Radiotherapy	Not generally	Risk of miscarriage [81]	Preterm birth, low birthweight, and stillbirth [82] Neurodevelopmental changes, future development of malignancy [81]	Fetal exposure varies based on location of primary tumor [83] Impact on fetus depends on gestational stage	Dependent on gestational age and primary tumor location
Chemotherapy	Yes, for the majority of antineoplastics *	Maternal infections, anemia [59] Long-term side effects depending on chemotherapy agent [84] Infertility [85]	Increased teratogenicity when given in first trimester [86] Slight increased risk of low birth weight and/or premature delivery [59] Risk of neonatal hematologic sequelae and myelosuppression if given within 3–4 weeks of delivery [76,87]	Low birth weight and/or premature delivery may also be related to underlying disease severity Impact on fetus when initiated in early second trimester	Inclusion of cardiologists to survey possible cardiotoxicity Treatment pause recommended within 3–4 weeks of delivery (at 34–36 weeks gestation if at-term delivery is anticipated) to avoid neonatal myelosuppression Treatment should mimic that for non-pregnant patients, using weight-based dosing [88] Hypervolemia, enhanced renal/hepatic elimination and reduced albumin in pregnancy may impact pharmacokinetics [21] Inclusion of neonatologists in case of preterm delivery and low birth weight
Immunotherapy	No	No available human trials, little retrospective clinical data [89,90]	Fetal rejection, premature delivery, and infant mortality in animal models [89]	Not available	Research recommendation to further study kinetics and fetal exposure
Targeted therapy during pregnancy	Not generally	Risk of miscarriage, particularly within first trimester [91] Risk of oligo/ anhydramnios with trastuzumab [92] Teratogenicity and pregnancy loss with first-trimester use of tyrosine kinase inhibitors [91]	Many subtypes are teratogenic [93]	Care should be individualized depending on recommended targeted therapy	Research recommendation to further study kinetics and fetal exposure
Radiotherapy, Chemotherapy, Immunotherapy, and Targeted therapy during lactation	Not applicable	Lactation generally contraindicated given neonatal risk/benefit profile	Unknown level of infant exposure via breast milk [94] Unclear impact on infant development after exposure	Patients receiving external beam radiation may be able to breastfeed from the contralateral breast [95]	Research recommendation to further study kinetics and infant exposure
Supportive treatments for chemotherapy side effects	Yes, specifically antiemetics and systemic steroids	Hyperglycemia and increased maternal blood pressure with chronic systemic steroid administration [96]	Ondansetron-related teratogenicity demonstrated in original retrospective reviews (specifically, cleft palate) has not been confirmed in larger studies [96]	Non-fluorinated steroids, such as methylprednisolone, are the preferred first-line steroids over betamethasone or dexamethasone, as these agents do not cross the placenta [96]	Antiemetics and systemic steroids are considered safe in pregnancy if indicated for chemotherapy side effects
Iatrogenic pre-term delivery	No	Maternal stress and anxiety [97] Association with lifetime development of cardiovascular disease [98]	Complications of prematurity, increased neonatal morbidity [99,100]	Not available	Term delivery is recommended with goal for vaginal delivery, where obstetrically appropriate Neonatologists should be involved on conversations in case of spontaneous preterm delivery and/or low birthweight
Pregnancy termination	No	Generally, not medically recommended, no clear prognostic benefit to patient [101]	Termination of a possibly non-anomalous gestation	Those generally associated with pregnancy termination	Pregnancy continuation is at the discretion of the patient and contingent on national and local jurisdiction Certain instances of cervical cancer, acute leukemia at an early [83] gestational age or metastatic malignancies in which pregnancy termination may be necessary [102]

Footnotes: * Certain antineoplastic agents, such as methotrexate, are absolutely contraindicated in pregnancy.

## Data Availability

This manuscript is a perspective based on previously published studies; no original datasets were generated or analyzed. Therefore, data sharing is not applicable.

## Supplementary Materials

The following supporting information can be downloaded at https://www.mdpi.com/article/10.3390/curroncol33050296/s1, Table S1. Summary of clinical and research recommendations to improve the diagnosis, management, and understanding of cancer during pregnancy. Content represents an original synthesis by the authors based on the literature and expert discussions and does not reproduce or adapt previously published tables or figures. Table S2. Curated resources for clinicians, researchers, and patients related to cancer during pregnancy, including clinical guidelines, registries, research databases, patient support organizations, and additional initiatives. Content represents an original compilation by the authors.

## Abbreviations

NIH	National Institutes of Health
PRGLAC	Task Force on Research Specific to Pregnant Women and Lactating Women
ABCIP	Advisory Board on Cancer, Infertility and Pregnancy
INCIP	International Network on Cancer, Infertility and Pregnancy
ASCO	American Society of Clinical Oncology
ESGO	European Society of Gynaecological Oncology
MRI	Magnetic Resonance Imaging
PET/CT	Positron Emission Tomography/Computed Tomography
CT	Computed Tomography
CA 15-3	Cancer Antigen 15-3
SCCA	Squamous Cell Carcinoma Antigen
CA-125	Cancer Antigen 125
hCG	Human Chorionic Gonadotropin
AFP	Alpha-fetoprotein
cfDNA	Cell-free DNAdiss
BRCA1/2	Breast Cancer Gene 1/Breast Cancer Gene 2
mGy	Milligray (radiation dose unit)
