# Supplementary material for: Cancer During Pregnancy: Navigating Clinical and Research Challenges
Source: Curr Oncol. 2026 May 19;33(5):296. doi: 10.3390/curroncol33050296 (PMC13204943; doi:10.3390/curroncol33050296)
Supplement: Supplementary file 1 [file curroncol-33-00296-s001.zip › curroncol-4208076-supplementary.pdf]

Title: Cancer During Pregnancy: Navigating Clinical and Research Challenges

Authors: Mackenzie K. Callaway[1#], Lizelle Comfort[2#], Dhivyaa Anandan[1,3], Ruby Sharma[4], Narjust Florez[5], Traci R. Lyons[6,7], Doris Germain[8], Kathleen R. Cho[9], Burton L. Rochelson[2], Clarissa Bonanno[2], Kutluk Oktay[10,11], Sudarshana Roychoudhury[12], Eileen O'Donnell[4], Richard Barakat[4], Joanne Marquardt[13], Diana W. Bianchi[14,15], Elyce Cardonick[16], Larry Norton[17], Ann H. Partridge[5], Susan M. Domchek[18,19], Virginia F. Borges[6,7], Frédéric Amant[20,21], Camila O. dos Santos [1*]

Affiliations:

[1] Cold Spring Harbor Laboratory, Cancer Center, Cold Spring Harbor, NY, 11724 USA.
[2] Department of Obstetrics and Gynecology, Division of Maternal Fetal Medicine, Northwell Health and Donald and Barbara Zucker School of Medicine at Hofstra/Northwell, Northwell Health, New Hyde Park, NY, 11042 USA.
[3] Stony Brook Medical Scientist Training Program, Stony Brook, NY, 11790 USA.
[4] Northwell Health Cancer Institute, 1111 Marcus Avenue, New Hyde Park, NY, 11042 USA.
[5] Dana-Farber Cancer Institute, Harvard Medical School, Boston, MA 02215, USA.
[6] Department of Medicine, Division of Medical Oncology, University of Colorado Anschutz Medical Campus, Aurora, CO 80045, USA.
[7] University of Colorado Cancer Center Young Women's Breast Cancer Translational Program, Aurora, CO 80045, USA.
[8] Icahn School of Medicine at Mount Sinai, Department of Medicine, Tisch Cancer Institute, New York, NY 10029, USA.
[9] Department of Pathology, University of Michigan Medical School, Ann Arbor, MI 48109, USA.
[10] Department of Obstetrics, Gynecology and Reproductive Sciences, Yale School of Medicine, New Haven, CT 06510, USA.
[11] Innovation Institute for Fertility Preservation, New York, NY 10016, USA.
[12] Department of Pathology and Laboratory Medicine, Donald and Barbara Zucker School of Medicine at Hofstra/Northwell Health, Greenvale, NY 11549, USA.
[13] Babylon Breast Cancer Coalition, 218N Wellwood Ave Suite #2, Lindenhurst, NY 11757, USA.
[14] Prenatal Genomics and Therapy Section, Center for Precision Health Research, National Human Genome Research Institute, National Institutes of Health, 35A Convent Drive Bethesda, MD 20892, USA.
[15] Eunice Kennedy Shriver National Institute of Child Health and Human Development, National Institutes of Health, 31 Center Drive, Bethesda, MD 20892, USA.
[16] Department of Obstetrics and Gynecology, Cooper University Health Care, Camden, NJ 08103, USA.
[17] Department of Medicine, Memorial Sloan Kettering Cancer Center, New York, NY 10065, USA.
[18] Department of Medicine, University of Pennsylvania Health System, Philadelphia, PA 19104, USA.
[19] Baser Center for BRCA, Abramson Cancer Center, University of Pennsylvania, Philadelphia, PA 19104, USA.
[20] Department of Oncology, KU Leuven, Leuven, Belgium; Department of Obstetrics and Gynecology, UZ Leuven, Leuven, Belgium.
[21] Gynecologic Oncology, Antoni van Leeuwenhoek, Amsterdam, the Netherlands.

[#] These authors contributed equally to this manuscript

***Correspondence:** Camila dos Santos - dossanto@cshl.edu

Keywords: Cancer During Pregnancy, maternal-fetal care, cancer, health disparity

Supplementary Tables

Table S1. Summary of clinical and research recommendations to improve the diagnosis, management, and understanding of cancer during pregnancy. Content represents an original synthesis by the authors based on the literature and expert discussions and does not reproduce or adapt previously published tables or figures.

	**Clinical Recommendations**	**Research Recommendations**
**1**	Incentivize discussions between providers (OBGYN) and pregnant patients regarding evidence-based information on cancer-related symptoms.	Expand the investigation of medical imaging analysis and physiological changes in pregnant patients with cancer to improve screening and monitoring of high-risk patients.
**2**	Expand awareness and promote urgency in investigating and evaluating persistent and/or atypical symptoms during pregnancy, including when clinical results are inconclusive.	Generate new model systems and genomic datasets to provide a scientific basis for the selection of markers that could then be investigated utilizing imaging or non-invasive liquid biopsy diagnostic approaches.
**3**	Form care teams early in the diagnostic process to streamline evaluation and treatment planning.	Incentivize the inclusion of pregnant patients in clinical trials, and models of pregnancy in basic research.
**4**	For most cancers, consider the use of whole-body diffusion-weighted MRI without gadolinium as first-line imaging when non-breast primary malignancy is suspected. Consider the potential use of oral manganese-containing contrast agents, such as pineapple juice, for enhancing MRI imaging—recognizing that this remains an emerging technique not yet widely adopted in clinical practice.	Promote multi-center, global, and data-driven studies by the scientific and medical communities to generate comprehensive databases of pregnant patients with distinct types of cancers.
**5**	Connect and consult with networks such as ABCIP and INCIP to improve diagnostics and patient standard-of-care.	Create multidisciplinary teams for diagnosing, treating, and supporting patients diagnosed with cancer in pregnancy.
**6**	Build multidisciplinary care teams including OBGYNs, oncologists, surgeons, cardiologists, pathologists, reproductive endocrinologists, geneticists, neonatologists, radiologists, psychologists, and other additional expertise as needed to care for pregnant patients with cancer.	Incentivize the collection of comprehensive patient information including age, gestational age at diagnosis, obstetrical and gynecologic history, parity history, lactation history, offspring age, tumor subtype and stage, symptoms which led to cancer diagnosis, and known cancer risk factors.
**7**	Limit use of PET/CT to clinically necessary cases; avoid abdominal shielding due to potential harm.	Profile pharmacokinetics of therapeutic agents including chemotherapy, assess the propensity of such agents for placental transfer, entrance into fetal circulation, and transmission through breast milk into infant circulation.
**8**	Continue disseminating consensus guidelines on delivery and treatment outcomes for pregnant patients with cancer.	Support biobank creation and research incentives to advance knowledge of pregnancy-associated cancers.
**9**	Include fertility preservation discussions in patient counseling for pregnant individuals diagnosed with cancer.	Combine databases and communicate across centers, particularly for low-incidence cancers, to monitor maternal-fetal response to treatment strategies.
**10**	Develop survivorship programs for both patients and children exposed to cancer therapies *in utero.*	Develop long-term studies to follow up regarding cancer recurrence postpartum, and the outcomes for children exposed to cancer therapies *in utero.*

Table S2. Curated resources for clinicians, researchers, and patients related to cancer during pregnancy, including clinical guidelines, registries, research databases, patient support organizations, and additional initiatives. Content represents an original compilation by the authors.

Type	Resources	Resource link
Clinical Guidelines	Advisory Board on Cancer, Infertility and Pregnancy (ABCIP)	https://www.ab-cip.org/home
	European Society of Gynaecological Oncology (ESGO)	https://www.esgo.org/
	European Society for Medical Oncology (ESMO)	https://www.esmo.org/
	American Society of Clinical Oncology (ASCO)	https://www.asco.org/
Clinical Registries	International Network on Cancer, Infertility and Pregnancy (INCIP)	https://www.esgo.org/network/incip/
	Cancer and Pregnancy Registry (longitudinal study)	https://cancerandpregnancy.com/
Research Repositories	Drugs and Lactation Database (LactMed®)	https://catalog.data.gov/dataset/lactmed-09a41/resource/ff4894fa-92cd-41ca-8396-8bcd412368ba
	All of Us: database with thousands of studies on a variety of health conditions	https://allofus.nih.gov/
Patient Resources	Hope for Two (Support groups)	https://hopefortwo.org/
	Mummy's Star (Support groups)	https://www.mummysstar.org/
	Polite Tumor (Financial assistance)	https://www.politetumor.com/
	International Network on Cancer, Infertility and Pregnancy (INCIP, Patient platform)	https://cancerinpregnancy.org/patients/
	Breast Cancer in Young Women Foundation	https://breastcancerinyoungwomen.org/
	National Breast Cancer Coalition (NBCC)	https://www.stopbreastcancer.org/
	Young Breast Cancer Survivors Program	https://www.cdc.gov/comprehensive-cancer-control/related-programs/young-breast-cancer-survivors.html
	Young Survivors Coalition	https://youngsurvival.org/
Additional Resources	Coalition to Advance Maternal Therapeutics	https://safemeds4moms.org/
	Maternal and Pediatric Precision in Therapeutics Hub (MPRINT)	https://www.mprint.org/
	Task Force on Research Specific to Pregnant Women and Lactating Women (PRGLAC)	https://swhr.org/health_focus_area/maternal-and-infant-health-disparities/
